# An elusive diagnosis: *Scedosporium apiospermum* infection after near-drowning

**DOI:** 10.4103/0972-2327.70878

**Published:** 2010

**Authors:** Malini Gopinath, Ajith Cherian, Neeraj N. Baheti, Abhijit Das, Molly Antony, C. Sarada

**Affiliations:** Department of Neurology, Sree Chitra Tirunal Institute for Medical Sciences and Technology, Trivandrum, Kerala, India; 1 Department of Microbiology, Sree Chitra Tirunal Institute for Medical Sciences and Technology, Trivandrum, Kerala, India

**Keywords:** Fungal meningitis, near-drowning, *Pseudallescheria boydii*, *Scedosporium apiospermum*

## Abstract

A 51-year-old male was admitted in our institute following an episode of near-drowning. He later developed ventriculitis and cerebral ring-enhancing lesions. He died following a subarachnoid hemorrhage due to rupture of a mycotic aneurysm involving the right fetal posterior cerebral artery. *Scedosporium apiospermum* was isolated from the cerebrospinal fluid. Central nervous system invasion by *S apiospermum* may present insidiously in near-drowning patients and, therefore, requires a high index of suspicion. In cases with the characteristic cerebral ring-enhancing lesions and concomitant ventriculitis, treatment should be instituted while awaiting fungal culture. With this article we intend to alert neurologists, intensivists, and physicians to this near fatal infection, as early identification and prompt treatment with voriconazole may be life saving.

## Introduction

Central nervous system (CNS) infection is an uncommon but serious complication of near-drowning. *Scedosporium apiospermum* and its sexual form *Pseudallescheria boydii*, a soil and waterborne fungus, has been incriminated as a common causative agent in this setting.[1,2] These infections are usually insidious in onset and typically occur a few weeks after the initial submersion injury.[3] CNS invasion by *S apiospermum* is resistant to amphotericin B and is very often fatal.[4] We report the case of an adult male who died following subarachnoid hemorrhage after a near-drowning episode. Culture of the cerebrospinal fluid (CSF) later showed growth of *S apiospermum*.

## Case Report

**Figure 1:**
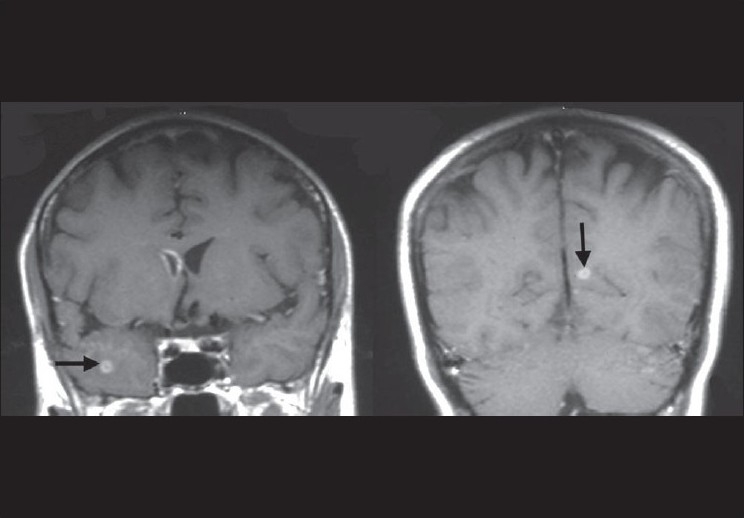
MRI coronal postcontrast T1 sequence shows ringenhancing lesion in the right anterior temporal pole and left parietooccipital region (black arrows); there is enhancement involving the right frontal horn, suggesting ventriculitis

A 51-year-old farmer had a near-drowning episode and was resuscitated after rescue. He developed bronchopneumonia and was managed with parenteral antibiotics. Two weeks later he developed bifrontal headache, with features suggestive of raised intracranial pressure. Examination showed neck stiffness and a positive Kernig sign, without other focal neurological deficits. Magnetic resonance (MR) imaging of the brain showed ring-enhancing lesions in the left parieto-occipital region, right anterior temporal pole, and left temporal lobe. In addition, ventriculitis involving the right frontal horn was noted [Figure 1]. CSF examination showed 254 cells/mm^3^ with 65% polymorphs and 35% lymphocytes. CSF glucose was 43 mg/dl (CSF glucose/plasma glucose ratio: 0.53) and protein 58 mg/dl. Gram’s stain, acid-fast bacillus stain, and India ink stain of the CSF were all negative, so was CSF cysticercal antibody and panfungal antigen assay. CSF culture for pyogenic bacteria and tubercle bacilli and blood cultures were sterile. CSF-PCR for tuberculosis was also negative. Fungal culture reports were awaited.

**Figure 2:**
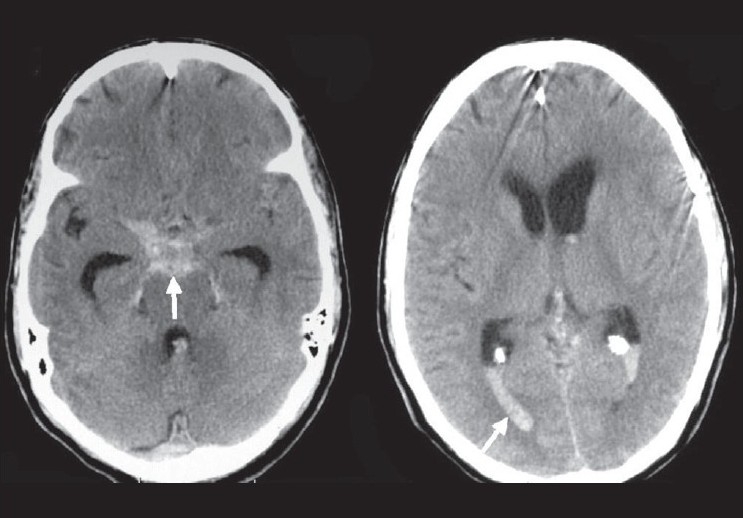
Computerized tomography (CT) shows diffuse subarachnoid hemorrhage (SAH) involving the basal cisterns, prepontine cistern (white arrow), left sylvian cistern, bilateral occipital horns (white arrow), third and fourth ventricle along with mild hydrocephalus

**Figure 3:**
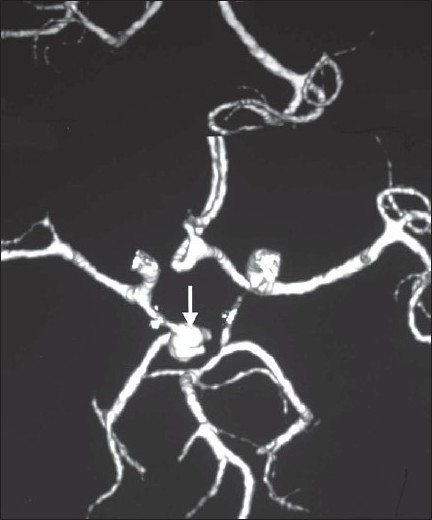
Three-dimensional reconstructed CT angiogram shows fusiform aneurysm with a saccular projection arising from the middle of right fetal posterior cerebral artery (PCA) and directed posterosupero- laterally (white arrow)

Subacute meningitis was considered as a diagnostic possibility as the patient had had prior bronchopneumonia and he was started on empirical broad-spectrum antibiotics in meningitic dose. He continued to be febrile without demonstrating any focal deficits. Six weeks into the illness, he developed recurrent seizures and a repeat computerized tomography (CT) of the brain showed a subarachnoid hemorrhage (SAH) involving the basal cisterns and cortical sulci; extending to the occipital horns, third and fourth ventricles. There was also mild hydrocephalus. [Figure 2]. CT angiogram showed an 8 × 8 mm fusiform aneurysm with a saccular projection arising from the middle of the right fetal posterior cerebral artery (PCA), directed postero-supero-laterally [Figure 3]. The patient’s condition deteriorated abruptly, precluding any invasive management. Despite supportive measures, he succumbed to the illness two months after the near-drowning episode. *Scedosporium apiospermum* in Graphium state (asexual coremial form) was later grown from the CSF sample sent for fungal culture.

## Discussion

Scedosporium species belongs to the filamentous fungi. Two species, *S apiospermum* and *Scedosporium prolificans* have health effects that can be serious and even fatal. The telemorph (sexual phase) of *S apiospermum* is called *Pseudallescheria boydii*. *S apiospermum* has yet another asexual coremial form called *Graphium eumorphum*, but this is usually reported as the ‘Graphium state of *Scedosporium apiospermum*.’[1,2]

*S apiospermum* is a ubiquitous fungus of soil and polluted water.[2] It causes a wide spectrum of diseases, from localized to invasive infections in both immunocompetent and immunocompromised patients. The most common presentation is as white grain mycetoma, a chronic subcutaneous fungal infection of the lower extremities. *S apiospermum* can also invade the lungs, kidneys, thyroid, eye, and brain.[1,3] CNS invasion may be by direct inoculation (during trauma or surgery), by extension from a source near the brain (paranasal sinuses, eye, or ear), or by hematogenous spread from the lung in cases of massive aspiration (as in near-drowning). There is increasing evidence that aspiration of polluted water is an important factor predisposing to CNS infection, even in immunologically competent patients.[4,5] The majority of cases that have been reported following near-drowning incidents have shown clinical evidence of pulmonary infection (with diffuse or localized lung infiltrates on chest X-ray) prior to CNS dissemination; as was true in our case also.[6], The CNS manifestation is usually insidious in onset, typically occurring a few weeks after the initial submersion injury.[5]

CNS infection can manifest as brain abscess, meningitis, encephalitis, ventriculitis, or as vascular involvement with resultant sinus thrombosis and cerebral infarction.[1] Of the 24 cases of CNS invasion with *S apiospermum* following near-drowning (excluding this one), 17 had cerebral abscesses at presentation, with three patients having concomitant ventriculitis. Ventriculitis has been attributed to the spread of infection via the choroid plexus.[6–8] The initial MR imaging in our patient showed ventriculitis involving the right frontal horn, in addition to multiple ring-enhancing lesions.

Like Aspergillus species, *S apiospermum* shows a high affinity for blood vessels. It quickly invades the cerebral vessels, causing ischemia and brain infarction.[8,9] Concomitant intracerebral, intraventricular, or subarachnoid hemorrhages secondary to angioinvasion have been reported rarely.[9,10] Cerebral aneurysms caused by this organism are even rarer. They usually affect the circle of Willis and the proximal arterial tree, tend to enlarge and extend, involving long segments of the vascular wall. Pathologic studies have shown that they are friable and poorly defined. In our case, the mycotic aneurysm was detected only after subarachnoid hemorrhage had occurred. Endovascular or surgical treatment of these aneurysms is extremely difficult, if not impossible. They are associated with a very high (nearly 100%) mortality rate once they rupture.[11,12]

Early diagnosis of the infection may be difficult as the onset of neurological symptoms revealing CNS invasion may be delayed (by several weeks or even months), as in our case.[4,5] A high index of suspicion should be maintained in near-drowned patients to ensure early diagnosis of *S apiospermum* infection and detection of CNS dissemination.[8]

CNS infection caused by *S apiospermum* is uniformly fatal without treatment. Even with surgical intervention and antifungal therapy, three-fourths of the patients do not survive.[6] Complicating the issue further, *S apiospermum* has often turned out to be resistant to a variety of conventional antimycotic agents, including amphotericin B.[4,8,12–14] Voriconazole is the agent that best combines the qualities of anti-scedosporial activity and CNS penetration. Prolonged treatment (>1 year) with voriconazole may lead to resolution of brain and lung lesions and thus help avoid surgical resection.[15]

## Conclusion

Neurological symptoms due to *S apiospermum* may appear late in cases of near-drowning and the presence of unexplained fever or neurologic abnormalities in near-drowning victims should always raise suspicion for this infection. Cerebral ring-enhancing lesions and concomitant ventriculitis with or without preceeding pneumonia in this setting is virtually diagnostic. In this scenario, initiating therapy with voriconazole while awaiting fungal isolation may be life saving.
